# Prevalence of Social Media Addiction and Its Determinants Among College Students in Chengalpattu District, Tamil Nadu

**DOI:** 10.7759/cureus.84625

**Published:** 2025-05-22

**Authors:** Gokul Gopakumar, Hariharan Surathkumaar, Ramkumar T, Aljin V, Subhashini Viswanath, Jeffrey Joseph

**Affiliations:** 1 Community Medicine, Sree Balaji Medical College & Hospital, Bharath Institute of Higher Education and Research, Chennai, IND; 2 Community Medicine, Meenakshi Medical College Hospital and Research Institute, Meenakshi Academy of Higher Education and Research (Deemed to be University), Kanchipuram, IND

**Keywords:** behavioral change, internet addiction disorder, personal relationship problems, self-esteem, young youths

## Abstract

Introduction

A persistent desire to use social media platforms is an indication of social media addiction, which has an adverse impact on academic performance, mental health, and social connections. According to WHO statistics, the percentage of adolescents using social media increased from 7% in 2018 to 11% in 2022. Social media improves communication among college students, but it can also lead to misuse. Addiction affects 18.4% of students worldwide, with higher percentages in Asia. Around 36.9% of college students in India exhibit addiction-related behaviors that are connected to anxiety, eye strain, poor sleep, and decreased academic performance. Addiction trends are further influenced by location and gender disparities. In Tamil Nadu, social media is widely used; however, little is known regarding its consequences. The purpose of this study is to determine the prevalence of social media addiction and its related factors among college students in the Chengalpattu district.

Methodology

A cross-sectional study was performed among students pursuing professional courses in the Chengalpattu district. A total of 320 participants were selected using simple random sampling. Data were collected using the Bergen Social Media Addiction Scale and the Rosenberg Self-Esteem Scale and analyzed using Statistical Package for Social Sciences (SPSS) version 25 (IBM Corp., Armonk, NY). Descriptive statistics were used to present data in the form of tables, and the p-value was calculated, and logistic regression analysis was performed.

Results

Among 320 students, 253 (79.1%) were in the age group of 18-21. Of those, 116 (36.3%) were male and 204 (63.7%) were female. A total of 243 (75.9%) participants were from nuclear families and resided in cities. For at least one to four hours per day, the majority of them used Instagram as their primary social media channel, followed by YouTube and others. Of the participants, it was found that 165 (51.6%) had good self-esteem, 154 (48.4%) had low self-esteem, and 18 (5.6%) were addicted to social media. Among the participants who have been surveyed, 42 (13%) reported that their personal relationships were affected because of social media, and they were much more likely to experience relationship problems (adjusted odds ratio (AOR): 4.69, 95% confidence interval (CI): 1.18-7.54, p=0.027). Around 36 (11.3%) individuals with social media addiction said they used social media for more than three hours per day. People who spent more than three hours a day were substantially more likely to be addicted to social media than those who spent less than that time (AOR: 4.71, 95% CI: 1.12-7.14, p=0.023).

Conclusion

According to this study, college students are becoming more involved in social media, with adolescents being the most active users. Usage may be supported by towns and nuclear families. Long-term use is linked to addictive behaviors and difficult relationships, despite the low overall rates of addiction. Similar levels of self-esteem suggest different psychological effects, emphasizing the necessity of mental health awareness and support to promote better use.

## Introduction

Social media has become an integral part of today's life, mostly among college students, and it is used to facilitate social interaction, share information, and initiate communication. But excess usage has raised concerns about social media addiction, which is defined as a constant desire to use social media and has a detrimental effect on social interactions, mental health, and academic performance. With an emphasis on recent developments and factors among college students, this introduction analyzes the prevalence of social media addiction globally, regionally in India, and in Tamil Nadu. 18.4% of students worldwide suffer from social media addiction, with Asia having the highest prevalence at 22.8%, according to a meta-analysis and comprehensive review [1]. According to the WHO Regional Office for Europe, the percentage of adolescents who use social media has increased significantly, from 7% in 2018 to 11% in 2022 [2]. This shows that excessive use of social media may be influenced by geographical variables, including educational needs, cultural standards, and access to the internet. Major issues are raised by this growing concern regarding how digital technology affects the general and mental health of adolescents. Furthermore, 12% of adolescents are susceptible to problematic gaming, which can lead to increased anxiety, poor academic performance, and social disengagement. More than 10% of adolescents engage in problematic social media behaviors, which are classified by compulsive use, low self-esteem, and detrimental psychological consequences. Unexpectedly, girls reported greater concerns about their social media use than boys, suggesting that there may be differences among genders in the probability of developing a digital addiction and experiencing adverse mental health impacts [2].

Prevalence estimates from another study that examined data from 32 nations showed significant regional variations, ranging from 0% to 82%. These variations illustrate how social, cultural, and technological elements influence the prevalence of social media addiction [3]. Developing region-specific prevention strategies to reduce the negative consequences of students' excessive use of social media might be made easier with an understanding of these variances.

The sudden rise of smartphones and internet access in India has raised concerns about college students' addiction to social media. The frequency of internet addiction among Indian college students was 40.7%, according to a study that examined the issue, highlighting just how common it is. According to a recent study on social media addiction, 36.9% of users engaged in addictive behaviors, which frequently had a negative impact on their well-being [4]. Problems with sleep (26.1%), anger (25.5%), and eye strain (38.4%) are common issues related to excessive social media use, suggesting both psychological and physical consequences [5]. According to these findings, a significant percentage of Indian college students may be at risk of being addicted to social media, highlighting the urgent need for awareness campaigns and focused intervention techniques to mitigate its effects.

People, especially young people, have been greatly impacted by social media, which has affected their interactions and behaviors. Adolescents who have low self-esteem are especially at risk, and they frequently use social media as a coping mechanism. Over time, this excessive use can result in social media addiction, where individuals feel compelled to stay connected, constantly checking updates, likes, and messages. Addiction often begins with a particular platform that serves as a gateway to problematic internet use, and this excessive engagement disrupts academic performance, emotional well-being, and real-life social interactions. Social media addiction is characterized by an overwhelming urge to stay online, neglecting relationships and responsibilities [6].

On average, Indians spend 2.4 hours a day on social media. Young people between the ages of 18 and 24 years use Facebook and Instagram at exceptionally high rates, with 97.2 million and 69 million users, respectively [2]. Using social media can lead to dopamine-driven addiction. The feedback loop mechanisms of the dopamine reward system keep users interested. One of the main characteristics of social media addiction is excessive and compulsive use of social media. Overuse of social media can be harmful in a variety of ways and interfere with other facets of a person's life. Increased dependence on social media as a coping strategy, detrimental effects on one's personal life, and restlessness while unable to check social media are all indicators of social media addiction [7,8].

Overuse of social media impacts mental health, sleep patterns, and cognitive abilities. It also changes brain reward pathways, resulting in addictive behaviors akin to substance abuse. Few studies have looked at the addiction patterns and influencing factors of social media use in India, particularly in Chengalpattu district, despite the fact that college students use social media more frequently. This study aims to bridge this gap by identifying prevalence, determinants, and potential interventions.

Excessive use of social media by Indian college students has been associated with a number of adverse consequences, including decreased sleep, poor academic performance, mental health issues, and physical health issues. According to a study of medical students in Gujarat, 32.1% of the students became dependent on social media, which was strongly associated with higher stress, anxiety, and depressive symptoms. Another major concern is sleeping difficulties, as the research performed among Delhi's undergraduate medical students found that excessive social media use was linked to both shorter sleep duration and lower sleep quality. Regular use of social media has been associated with poorer performance on university exams, suggesting that it affects students' ability to concentrate and be productive. The findings demonstrate how urgent measures are needed to control students' use of social media [9-11].

Indian college students have developed severe health and behavioral issues as a consequence of prolonged screen time caused by excessive social media use. Around 38.4% of students in Bengaluru study cited physical medical conditions such as eye strain, whereas 25.5% observed behavioral changes, including higher dissatisfaction and a lack of patience. Likewise, a research project performed among medical students in Gujarat found that 27.5% of them suffer from social media addiction, which raises their susceptibility to cyberbullying. Anxiety and stress are two mental health issues that cyberbullying can exacerbate. To decrease excessive social media use and minimize its negative effects on students' physical, emotional, and psychological well-being, our findings emphasize the urgent need for targeted measures and awareness campaigns [5,10]. Since Chengalpattu is a rapidly developing district, college students are under high academic pressure and face a lot of personal challenges due to urbanization and peer pressure, which drives students to seek stress relief through social media, making them more prone to addiction. Knowing the importance of this problem is vital for prompt response and policy formulation. The present study was undertaken to estimate the prevalence of social media addiction and its determinants among college students in Chengalpattu district.

## Materials and methods

The study was conducted in colleges across the Chengalpattu district, Tamil Nadu, among students pursuing professional courses. Data collection was carried out after obtaining permission from the respective institutions, and students above 18 years of age were included in the study. The sample size was calculated based on a study performed by Caner et al. [12], with the prevalence of social media addiction being 24.4%. Using this as prevalence (p) and substituting it in Dapson’s formula and adding a 10% non-response rate with an absolute precision of 5%, the final sample size of 320 was obtained. A line list was obtained from each college, and participants were selected using simple random sampling. Students were provided with a summary of the study's objectives and assurances of confidentiality. The study was conducted over four months, from November 2024 to February 2025. A total of 320 students participated after providing informed consent, while others either declined participation or were unavailable for data collection. Ethical committee approval was obtained from the Institutional Human Ethics Committee of Sree Balaji Medical College & Hospital (Approval No. 002/SBMCH/IHEC/2024/2328).

Data collection tools

Bergen Social Media Addiction Scale

A quick and efficient tool for evaluating the level of social media addiction, the Bergen Social Media Addiction Scale (BSMAS) was used to determine the prevalence of social media addiction. Each one of the six components gets a rating on a 5-point Likert scale, with scores ranging from 1 (which means "very rarely") to 5 (which means "very often"). These items assess the level of social media obsession, including the duration of time spent imagining about it or planning to use it, a need to use it more, the use of social media as an escape from personal issues, failed attempts to cut back on usage, nervousness or distress when not able to use it, and the negative effects of excessive use on people's capacity to work or study. The BSMAS score of 24 has been found to be the optimum cut-off point, consistent with the highest standards of clinical assessment. The total scores vary from 6 to 30, wherein a higher score suggests an elevated level of problematic social media use [12-14].

The socio-demographic information and other variables associated with social media addiction and self-esteem were collected using a pre-tested semi-structured questionnaire.

Rosenberg Self-Esteem Scale

Morris Rosenberg's 10-item Rosenberg Self-Esteem Scale (RSES), which was first developed for high school students, is now widely used to measure self-esteem across a range of groups of people. The Guttman scale approach, which combines responses to particular questions, and a summed 4-point scale with reverse-scored negative items are the two methods used for scoring. The RSES has remarkable reliability with a reproducibility coefficient of 0.92 and test-retest correlations between 0.85 and 0.88. Strong validity is demonstrated by the expected relationships it shows with measures of anxiety and depression as well as with other measures of self-esteem such as the Coopersmith Self-Esteem Inventory [15,16].

Data were entered in Microsoft Excel (Microsoft Corporation, Washington, United States) and analyzed using the Statistical Package for Social Sciences (SPSS) version 25 (IBM Corp., Armonk, NY). Descriptive statistics were used to present data in the form of tables. Analytical statistics like chi-square and odds ratio were used with statistical significance (at 95% CI) to assess the strength of association between various factors associated with social media addiction. Multiple logistic regression analysis was used to eliminate the confounders and find out the predictors of social media addiction.

## Results

Table 1 shows that 320 students studying in professional courses were studied. Among the study participants, 253 (80%) belong to the age group of 18-21 years. Of which 204 (63.7%) were female participants. Out of all the participants, 243 (75%) belong to a nuclear family. The majority of the participants were living in urban areas. Most of them used Instagram as the social media platform, followed by YouTube and others. Nearly half of them have been found using social media at least one to four hours per day.

**Table 1:** Sociodemographic data of the study participants (N=320)

Sl. no	Variable	Frequency (N)	Percentage (%)
1	Age (in years)		
	18-21	253	79.1
	22-25	67	20.9
2	Gender		
	Female	204	63.7
	Male	116	36.3
3	Type of family		
	Nuclear family	243	75.9
	Joint/three-generation family	77	24.1
4	Father’s occupation		
	Clerical/unskilled	51	15.9
	Professional/semi-professional	269	84.1
5	Residential area		
	Urban	298	95.5
	Rural	14	4.8
6	Social media platform		
	Instagram	263	84.2
	YouTube	40	12.8
	Snapchat/Facebook/Twitter	17	5.4
7	Hours spent on social media usage		
	Less than 1 hour	20	6.7
	1–2 hours	75	25.1
	2–3 hours	83	27.8
	3–4 hours	63	21.1
	More than 4 hours	58	19.4
8	Course of the students		
	Engineering	101	32.3
	Medical	138	44.2
	Law	73	23.3

**Figure 1:**
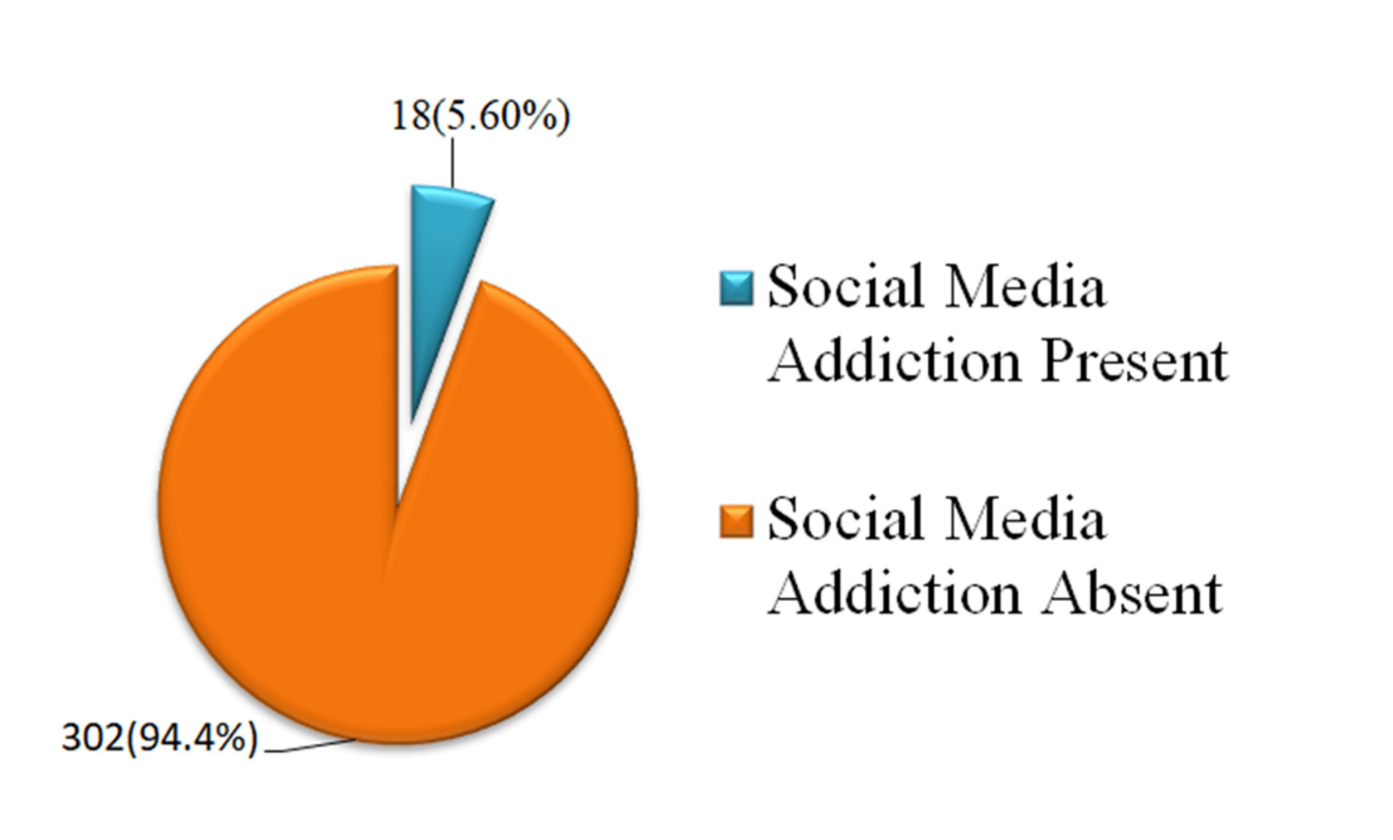
Prevalence of social media addiction among students

Figure 1 shows that out of 320 participants, 18 (5.6%) were addicted to social media, while 302 (94.4%) were not.

**Figure 2:**
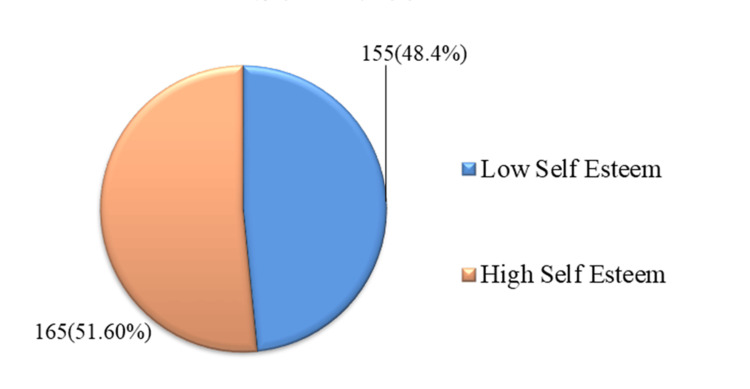
Pattern of self-esteem distribution among college students

Figure 2 shows that among the study participants, 155 (48.4%) have low self-esteem, while 165 (51.6%) have high self-esteem.

Table 2 shows that among the participants, 13% reported that social media use had negatively impacted their personal relationships. Individuals with social media addiction had significantly higher odds of experiencing relationship issues compared to those without addiction (adjusted odds ratio (AOR): 4.69, 95% confidence interval (CI): 1.18-7.54, p=0.027). This indicates that social media addiction is a significant predictor of relationship disturbances among college students.

Among participants with social media addiction, 11.3% reported spending more than three hours per day on social media. Compared to those who spent less than three hours daily, individuals exceeding this threshold had significantly higher odds of social media addiction (AOR: 4.71, 95% CI: 1.12-7.14, p=0.023). This suggests that prolonged social media use is a strong predictor of addiction among college students.

**Table 2:** Association of social media addiction with other determinantal factors *Represents p-value<0.05, which is considered statistically significant CI, confidence interval

S.No	Variable	Social media addiction				Chi square (x^2^)	Unadjusted odds ratio, (95% CI)	Adjusted odds ratio, (95% CI)	p-value
		Present		Absent					
		N	%	N	%				
1	Compelled to check social media accounts	11	12.2	79	87.8	10.23	4.43 ( 1.66-11.84)	-	0.143
2	Social media use has affected my personal relationships	13	13.0	87	87	14.85	6.42 (2.22-18.56)	4.69 (1.18-7.54)	0.027*
3	Hard to focus on daily activity due to social media use	11	8.5	118	91.5	3.41	2.45 (0.92-6.44)	-	0.144
4	Feeling anxious/stressed due to social media use	12	11.7	91	88.3	10.28	4.63 (1.68-12.73)	-	0.360
5	Positive impact on life due to social media use	6	3.0	197	97.0	7.42	0.26 (0.09-0.73)	-	0.258
6	Low self-esteem (Rosenberg questionnaire)	73	47.1	82	52.9	30.51	4.00 (2.28-6.64)	-	0.718
7	Average hours spent on social media								
	>3 hours	15	11.3	118	88.7	13.65	7.79 (2.20-27.51)	4.71 (1.12-7.14)	0.023*
	<3 hours	3	1.6	184	98.4				
8	No. of times social media used								
	Every few minutes/hours	14	7.8	165	92.2	3.6	2.90 (1.13-9.03)	-	0.472
	Once a day/more than once a day	4	2.8	137	97.2				

## Discussion

The addiction to social media is increasing among college students, which has an impact on their academic performance, social networks, and mental health. This study assesses its prevalence and associated characteristics among people in the Chengalpattu District of Tamil Nadu. The results suggest that significant behavioral patterns, risk factors, and demographic characteristics are responsible for excessive social media use. This discussion puts the findings in the context of previous studies, highlighting the need for targeted interventions and awareness campaigns to encourage good digital activities and ensure the well-being of students.

Of the participants in this study, 204 (63.7%) were female, and 253 (80%) were in the 18-21 age group. Among these, Instagram remained the most preferred social media site, followed by YouTube and other apps. Nearly half of the respondents, according to the study, used social media for one to four hours per day, and the rest of the participants used social media for more than four hours per day. Together with average daily usage, these trends show a strong preference for eye-catching and interactive applications among young adults, suggesting social media plays a significant part in their daily lives for communication, entertainment, and perhaps educational purposes, and 5.6% of the study participants have social media addiction. A study conducted on undergraduate students by the University of Albany found that 10% of social media users exhibited addictive behaviors [17]. Similarly, Salari et al. reported a prevalence of 18% for social media addiction [1]. In an urban area of Bangalore, a study found the prevalence to be 36.9% [5], while research among medical students in Saudi Arabia revealed a significantly higher prevalence of 55% [18,19].

The meta-analysis research conducted across 32 nations also showed a higher prevalence of social media usage [3]. Variations in social media prevalence trends can be attributed to a number of factors. Differences in measuring tools and classification standards are often the reason for research discrepancies. Cultural values also have significance; for example, nations that value freedom may use social media differently than others. Age, gender, and financial status constitute some of the demographic variables that affect usage patterns and the characteristics of the research population. University students may use social media more significantly than the general population. Regional changes, such as access to technology and regional traditions, further aggravate such disparities, highlighting the complex and multidimensional nature of social media use.

The results of the study on the addictive behavior of social media by Basu et al. align with our study, which found that many people use YouTube and Facebook. The same study also stated that participants used social media for about 11 hours, consistent with our study. The study conducted by Ramesh et al. [5] reported findings similar to those of Basu et al. [9]. These findings differ from those of our study, in which younger users, such as college students, tend to like Instagram, while individuals of age above teens favor Facebook; these disparities could be the result of variations in the demographic makeup of the study populations. The inconsistent results are also probably caused by variations in sample size and composition, as well as shifts in social media trends over time.

In the present study, 5.6% of participants were found to have social media addiction. This aligns with the global research findings, which report prevalence rates ranging from 5% to 25%, depending on factors such as age, culture, and level of internet accessibility [20]. Such consistency across regions highlights the universal nature of addictive behaviors linked to social media, driven by psychological mechanisms such as instant gratification and reward-seeking. Frequent exposure to idealized information on the internet frequently results in negative self-comparisons, leading to dissatisfaction. Additionally, excessive social media use is known to increase thoughts of anxiety and depression, which worsens self-esteem [21]. Studies have shown that interventions targeting emotional well-being, such as cognitive-behavioral therapy and self-esteem building programs, can be effective in reducing social media addiction [22], emphasizing the importance of mental health strategies in managing online behaviors These psychological effects have been demonstrated globally, irrespective of cultural context, and methods that focus on psychological well-being, such as cognitive behavioral methods and self-esteem enhancement, consistently reduce addiction.

In the present study, 13% of participants reported that social media use negatively impacted their personal relationships. Individuals with social media addiction experience relationship issues compared to those without addiction. Additionally, among participants with social media addiction, 11.3% reported spending more than three hours per day on social media. These results are in line with other studies by Al-Menayes et al. and Keles et al., who also found a significant relationship between interpersonal conflicts and excessive social media use. According to their findings, extended usage increased the risk of developing a social media addiction and the related social impairments [21,23]. The consistency among studies may be explained by the psychological dynamics of social media use, particularly the manner in which the brain's reward system functions. Regular social media use may trigger dopamine-driven behaviors that prolong excessive behavior and lead people to ignore their in-person relationships, which can harm relationships and connections in everyday life [21].

However, according to a 2019 study by Orben et al., there is not much or no direct impact in the relationship between social media use and well-being, including relationship quality. This disparity could result from cultural backgrounds, different measurement methods, or the complex structure of social media interactions [24]. Przybylski and Weinstein found in another study that the moderate use of social media can sometimes result in addiction or other detrimental effects. The various studies have different definitions for what is considered prolonged use, which suggests that one's personal characteristics and platform engagement are important factors to consider [25].

Strengths and limitations

The strength of the study is that it addresses relevant and emerging public health issues among youth, offering valuable insight into the prevalence and determinants of social media addiction, and the use of standardized questionnaire enhances the reliability of the findings. Since the study is focusing on college students, a high-risk group for digital overuse makes the results significant for targeted intervention. It also contributes regional data from Tamil Nadu, aiding local policy and awareness programs. However, the study has limitations, self-reported data may be influenced by recall bias. The findings were limited to college students in the Chengalpattu district and may not be generalized for all youth, and differences in internet access, smartphone usage, and academic pressure across institutions were not considered.

Recommendations

It is recommended that structured digital education initiatives that educate young people about social media usage in an appropriate manner and its psychological effects be put into place in accordance with the findings. Colleges ought to incorporate behavioral healthcare support services that highlight improving self-esteem and managing thoughts and feelings. Counseling services may assist students in managing their internet behavior and developing healthy coping mechanisms. Addiction risk may be reduced by restricting daily screen usage with self-monitoring tools and awareness initiatives. Social ties can also be strengthened through offline relationship-building courses and peer support groups. All things looked at, encouraging responsible and balanced social media use requires an integrated approach involving families, educators, and mental health specialists.

## Conclusions

This study provides valuable information about the trends and impacts of college students' use of social media. The adolescents and women appear to be more involved, probably because of social, emotional, and academic considerations. Urban environments and nuclear families might make it simpler to use the internet, which could affect how individuals behave online. Even while there does not seem to be a high prevalence of social media addiction overall, prolonged usage is significantly linked to addictive behaviors. Furthermore, social media addiction has been reported to have detrimental effects on interpersonal interactions, indicating that it may disrupt interpersonal relationships. The nearly equal levels of self-esteem point to conflicting psychological impacts and highlight the critical importance of immediate interventions. Reducing the risk of addiction while motivating healthier social media use can be achieved by promoting mental wellness services and knowledge about technology.
